# Taste hyposensitivity in Japanese schoolchildren

**DOI:** 10.1186/1472-6831-14-36

**Published:** 2014-04-11

**Authors:** Mari Ohnuki, Masayuki Ueno, Takashi Zaitsu, Yoko Kawaguchi

**Affiliations:** 1 Department of Oral Health Promotion, Graduate School of Medical and Dental Sciences, Tokyo Medical and Dental University, Tokyo, Japan

**Keywords:** Taste hyposensitivity, Schoolchildren, Taste test, Oral health status

## Abstract

**Background:**

There is some research on taste disorder/hyposensitivity in special groups such as the elderly or patients presenting with specific taste problems, however few studies have been conducted among young populations. The objectives of this study were to estimate the prevalence of taste hyposensitivity and to investigate the relationship between taste hyposensitivity and oral health status in Japanese schoolchildren.

**Methods:**

Subjects were 237 primary and 112 junior high school students in Saitama Prefecture, Japan. In total, 349 (boys: 181, girls: 168) students aged 6–15 years participated in the study. Oral examinations and whole-mouth taste tests using four tastes (sweet, salt, sour and bitter) solutions were conducted on the subjects. A subject who could not recognize the taste of the solution was defined as demonstrating hyposensitivity.

**Results:**

Hyposensitivity was observed in 6.3% of all subjects for sweet-taste, 14.3% for salt-taste, 20.9% for sour-taste and 6.0% for bitter-taste. The prevalence of sweet, sour and bitter-taste hyposensitivity decreased as the subjects’ grade advanced. In contrast, the prevalence of salt-taste hyposensitivity increased in 7^th^-9^th^ grade subjects. Furthermore, the prevalence of bitter-taste hyposensitivity was significantly higher in males than females among 1^st^-3^rd^ graders.

Taste hyposensitivity had little association with oral health status, such as decayed teeth, filled teeth, dental plaque, gingival status and tongue coating.

**Conclusions:**

In this study, taste hyposensitivity was observed in 6.0%-20.9% of the students. There was little association between taste hyposensitivity and oral health status. The current study implies that the factors affecting the taste hyposensitivity in children may different from those in the elderly. Therefore it is necessary to further investigate the causes of taste hyposensitivity among younger generation.

## Background

In Japan, reports on the prevalence of taste disorders have been increasing [1]. According to Ikeda *et al.,* the number of taste disorder patients who consulted with otorhinolaryngology doctors was approximately 240,000 per year, and the number was increasing every year [2]. Subjects of most studies on taste disorder/hyposensitivity are the elderly or patients referenced with specific taste disorders, and there are few reports about taste disorder/hyposensitivity among younger generations. It is reported that the changes in taste are related to oral health status such as dry mouth, tongue coating and taste buds destruction or loss in the elderly [3]. However there are few epidemiological studies on associations of taste hyposensitivity with oral health conditions, especially among younger populations such as school students.

Various taste tests exist with differing advantages and disadvantages. For example, the taste disc method is a precise tool but takes a long time to perform per person [1]. Therefore the taste disc method is useful within a clinic setting, but it is not suitable for epidemiological research. A whole-mouth method appears most efficient in screening for a large number of people.

The objectives of this study were to estimate the prevalence of taste hyposensitivity using the whole-mouth taste test for four basic tastes (sweet, salt, sour and bitter) and to investigate relationships between taste hyposensitivity and oral health status in schoolchildren.

## Methods

### Subjects

Subjects in this research were 237 primary (1^st^-6^th^ grade: boys: 121, girls: 116) and 112 junior high (7^th^-9^th^ grade: boys: 60, girls: 52) schoolchildren aged 6–15 years in Saitama Prefecture, Japan. In total, 349 students (boys: 181, girls: 168) participated in the research. Students were 1^st^ to 9^th^ grade (1^st^-6^th^ : elementary, 7^th^-9^th^ : junior high) schoolchildren. The research was implemented as part of school health promotion activities, therefore basically all students participated in the activity except for those who were absent. The information about this study was explained to all the students, and the consent was received from the subjects who agreed to participate in the study. The research was conducted in 2009 and the study proposal was approved by the Tokyo Medical and Dental University Ethics Committee (Approval No. 250).

### Oral health examinations

Oral health examinations were conducted by three trained and calibrated dentists. The examinations were performed by using a dental mirror and an explorer under the artificial light. Decayed, missing, and filled teeth were assessed according to the Japanese school dental examination procedure [4,5].

Oral hygiene and gingivitis were evaluated by examining the anterior teeth in both upper and lower arches. Oral hygiene condition was recorded with a dental plaque score ranging from 0 to 2 (0, no observable plaque; 1, less than one-third of anterior teeth with observable plaque; and 2, more than one-third of anterior teeth with observable plaque). Gingival condition was investigated with a gingival status score ranging from 0 to 2 (0, healthy gingiva; 1, slight gingivitis without dental calculus and 2, moderate gingivitis with dental calculus around one or more anterior teeth).

The tongue coating area or thickness was evaluated visually with the criteria described by Oho *et al.*[6]. The area of the tongue coating was defined as the extent from 0 to 3 (0, no observable tongue coating; 1, less than one-third of tongue dorsum with coating; 2, one-third to two-third of tongue dorsum with coating; 3, more than two-third of tongue dorsum with coating). Thickness of the tongue coating was defined as: 0 = No coating; 1 = Thin; 2 = Moderate; 3 = Thick.

### Taste tests

Taste tests were performed using a whole-mouth method [7]. A 3% sucrose solution was used as sweet-taste test, 0.4% sodium chloride as the salt-taste, 0.05% tartaric acid as the sour-taste, and 0.004% quinine as the bitter-taste. Since previous studies defined a person who could not recognize these concentrations of solution as having sweet-taste, salt-taste, sour-taste and bitter-taste hyposensitivity [7,8], the same concentrations were also used in this study.

A total of 1 mL of taste test solution was placed on the center of the tongue dorsum with a syringe. Immediately following the test, the students were asked to respond to whether or not they identified the taste tested. Between taste tests, the students rinsed their mouths with distilled water in order to eliminate the influence of the previous taste.

### Data analysis

Students were divided into three groups (1^st^-3^rd^, 4^th^-6^th^ and 7^th^-9^th^) by grade.

Associations between taste hyposensitivity and oral health status were analyzed by Χ^2^-test. All statistics were performed using SPSS15.0 (SPSS Japan, Tokyo, Japan) and p-values less than 0.05 were considered to be statistically significant.

## Results

### Oral health status

Table 1 shows the oral health status of the subjects by grade and gender. There was a significant difference in the distribution of tongue coating thickness by gender among 1^st^-3^rd^ graders (p < 0.05). A statistically significant difference in the distribution of gingivitis by gender was detected among 4 ^th^-6^th^ graders (p < 0.05). No significant differences were observed in other oral health items between boys and girls.

**Table 1:** Oral health status of subjects by grade and gender

**Grade**	**Variables**	**Category**	**Total**		**Boys**		**Girls**		**P-value**
			**n**	**%**	**n**	**%**	**n**	**%**	
	DT+dt	0	64	57.7	30	51.7	34	64.2	n.s.
		1+	47	42.3	28	48.3	19	35.8	
	FT+ft	0	44	39.6	20	34.5	24	45.3	n.s.
		1+	67	60.4	38	65.5	29	54.7	
	Plaque score	0	56	50.5	29	50.0	27	50.9	n.s.
		1+	55	49.5	29	50.0	26	49.1	
1^st^-3^rd^	Gingivitis	0	79	71.2	43	74.1	36	67.0	n.s
		1+	32	28.8	15	25.9	17	32.1	
	Area of tongue coating	0,1	53	47.7	31	53.4	22	41.5	n.s
		2+	58	52.3	27	41.5	31	53.4	
	Thickness of tongue coating	0,1	67	60.4	41	70.7	26	49.1	0.032
		2+	44	39.6	17	29.3	27	50.9	
	DT+dt	0	104	82.5	51	81.0	53	84.1	n.s
		1+	22	17.5	12	19.0	10	15.9	
	FT+ft	0	45	35.7	22	34.9	23	36.5	n.s
		1+	81	64.3	41	65.1	40	63.5	
	Plaque score	0	57	45.2	25	39.7	32	50.8	n.s
		1+	69	54.8	38	60.3	31	49.2	
4^th^-6^th^	Gingivitis	0	66	52.4	27	42.9	39	61.9	0.049
		1+	60	47.6	36	57.1	24	38.1	
	Area of tongue coating	0,1	41	46.0	47	31.7	21	33.3	n.s
		2+	85	54.0	13	68.3	42	66.7	
	Thickness of tongue coating	0,1	58	46.0	17	42.9	31	49.2	n.s
		2+	68	54.0	43	57.1	32	50.8	
	DT + dt	0	81	72.3	47	78.3	34	65.4	n.s
		1+	31	27.7	13	21.7	18	34.6	
	FT + ft	0	42	37.5	17	28.3	25	48.1	n.s
		1+	70	62.5	43	71.7	27	51.9	
7^th^-9^th^	Plaque score	0	23	20.5	11	18.3	12	23.1	n.s
		1+	89	79.5	49	81.7	40	76.1	
	Gingivitis	0	43	38.4	21	35.0	22	42.3	n.s
		1+	69	61.6	39	65.0	30	57.7	
	Area of tongue coating	0,1	68	60.7	35	58.3	33	63.5	n.s
		2+	44	39.3	25	41.7	19	36.5	
	Thickness of tongue coating	0,1	84	75.0	44	73.3	40	76.9	n.s
		2+	28	25.0	16	26.7	12	23.1	

### Taste hyposensitivity

Table 2 shows the results of taste tests. The prevalence of sweet-taste hyposensitivity were 10.8% (1^st^-3^rd^ grade), 4.8% (4^th^-6^th^ grade), 3.6% (7^th^-9^th^ grade) and 6.3% (total). The prevalence of salt-taste hyposensitivity were 16.2% (1^st^-3^rd^ grade), 8.7% (4^th^-6^th^ grade), 18.8% (7^th^-9^th^ grade) and 14.3% (total). The prevalence of sour-taste hyposensitivity were 28.8% (1^st^-3^rd^ grade), 20.6% (4^th^-6^th^ grade), 13.2% (7^th^-9^th^ grade) and 20.9% (total). The prevalence of bitter-taste hyposensitivity were 16.2% (1^st^-3^rd^ grade), 2.4% (4^th^-6^th^ grade), 0% (7^th^-9^th^ grade) and 6.0% (total).

**Table 2:** Prevalence of taste hyposensitivity by gender

	**Sweet-taste hyposensitivity**							**Salt-taste hyposensitivity**						
**Grade**	**Total**		**Boys**		**Girls**		**p-value**	**Total**		**Boys**		**Girls**		**p-value**
	**n**	**%**	**n**	**%**	**n**	**%**		**n**	**%**	**n**	**%**	**n**	**%**	
1^st^-3^rd^	12	10.8	7	12.1	5	9.4	n.s.	18	16.2	13	22.4	5	9.4	n.s.
4^th^-6^th^	6	4.8	2	3.2	4	6.3	n.s.	11	8.7	6	9.5	5	7.9	n.s.
7^th^-9^th^	4	3.6	4	6.7	0	0.0	n.s.	21	18.8	13	21.7	8	15.4	n.s
Total	22	6.3	13	7.2	9	5.4	n.s.	50	14.3	32	17.7	18	10.7	n.s
	**Sour-taste hyposensitivity**							**Bitter-taste hyposensitivity**						
**Grade**	**Total**		**Boys**		**Girls**		**p-value**	**Total**		**Boys**		**Girls**		**p-value**
	**n**	**%**	**n**	**%**	**n**	**%**		**n**	**%**	**n**	**%**	**n**	**%**	
1^st^-3^rd^	32	28.8	21	36.2	11	20.8	n.s.	18	16.2	14	24.1	4	7.5	0.021
4^th^-6^th^	26	20.6	15	23.8	11	17.5	n.s.	3	2.4	2	3.2	1	1.6	n.s.
7^th^-9^th^	15	13.2	8	13.3	7	13.2	n.s.	0	0.0	0	0.0	0	0.0	-
Total	73	20.9	44	24.3	29	17.3	n.s.	21	6.0	16	8.8	5	3.0	0.024

The prevalence of sweet-, sour- and bitter-taste hyposensitivity decreased as the schoolchildren’s grade advanced, while the prevalence of salt-taste hyposensitivity decreased up to 4^th^-6^th^ grade and increased in 7^th^-9^th^ grade (p < 0.05).

In total, there was a significant gender difference in the prevalence of salt- or bitter-taste hyposensitivity (p < 0.05). There were no significant differences between boys and girls in sweet- and sour-taste.

As for differences by grade (Table 3), there was a significant difference in the prevalence of sweet-taste hyposensitivity between 1^st^-3^rd^ and 7^th^-9^th^ graders (p < 0.05) and sour-taste hyposensitivity between 1^st^-3^rd^ graders and 7^th^-9^th^ graders (p < 0.05). In the prevalence of bitter-taste hyposensitivity, there were significant differences between 1^st^-3^rd^ graders and 4^th^-6^th^ graders (p < 0.01), and between 1^st^-3^rd^ graders and 7^th^-9^th^ graders (p < 0.01).

**Table 3:** Significance of taste hyposensitivity by grade

	**Sweet-taste hyposensitivity**				**Salt-taste hyposensitivity**		
**Grade**	**1**^**st**^**-3**^**rd**^	**4**^**th**^**-6**^**th**^	**7**^**th**^**-9**^**th**^	**Grade**	**1**^**st**^**-3**^**rd**^	**4**^**th**^**-6**^**th**^	**7**^**th**^**-9**^**th**^
1^st^-3^rd^	-	n.s.	0.041	1^st^-3^rd^	-	n.s.	n.s.
4^th^-6^th^	-	-	n.s	4^th^-6^th^	-	-	0.035
	**Sour-taste hyposensitivity**				**Bitter-taste hyposensitivity**		
**Grade**	**1**^**st**^**-3**^**rd**^	**4**^**th**^**-6**^**th**^	**7**^**th**^**-9**^**th**^	**Grade**	**1**^**st**^**-3**^**rd**^	**4**^**th**^**-6**^**th**^	**7**^**th**^**-9**^**th**^
1^st^-3^rd^	-	n.s.	0.004	1^st^-3^rd^	-	0.001	0.001
4^th^-6^th^	-	-	n.s	4^th^-6^th^	-	-	n.s.

As presented in Table 4, there was a significant association between sour-taste hyposensitivity and area of tongue coating in 4^th^-6^th^ graders (p < 0.05) in this study.

**Table 4:** Association between taste hyposensitivity and oral health status

			**Sweet-taste hyposensitivity**				**Salt-taste hyposensitivity**				**Sour-taste hyposensitivity**				**Bitter-taste hyposensitivity**			
			**+**		**–**		**+**		**–**		**+**		**–**		**+**		**–**	
**Grade**	**variables**	**Category**	**n**	**%**	**n**	**%**	**n**	**%**	**n**	**%**	**n**	**%**	**n**	**%**	**n**	**%**	**n**	**%**
	DT+dt	0	5	7.8	59	92.2	8	12.5	56	87.5	16	25.0	48	75.0	12	18.8	52	81.3
		1+	7	14.9	40	85.1	10	21.3	37	78.7	16	34.0	31	66.0	6	12.8	41	87.2
	FT + ft	0	4	9.1	40	90.9	9	20.5	35	79.5	16	36.4	28	63.6	4	9.1	40	90.2
		1+	8	11.9	59	88.1	9	13.4	58	86.6	16	23.9	51	76.3	14	20.9	53	79.1
1^st^-3^rd^	Plaque	0	7	12.5	49	87.5	13	23.3	43	76.8	16	28.6	40	71.4	12	21.4	44	78.6
		1+	5	9.1	50	90.9	5	9.1	50	90.9	16	29.9	39	70.9	6	10.9	49	89.1
	Gingivitis	0	9	11.4	70	88.6	14	17.7	65	82.3	27	34.2	52	65.8	13	16.5	66	71.0
		1+	3	9.4	29	90.6	4	12.5	28	87.5	5	15.6	27	84.4	5	15.6	27	29.0
	Area of tongue coating	0,1	6	11.3	47	88.7	10	18.9	43	81.1	17	32.1	36	67.9	9	17.0	44	83.0
		2+	6	10.3	52	89.7	8	13.8	50	86.2	15	25.9	43	74.1	9	15.5	49	84.5
	Thickness of tongue coating	0,1	7	10.4	60	89.6	13	19.4	54	80.6	21	31.3	46	68.7	13	19.4	54	80.6
		2+	5	11.4	39	88.6	5	11.4	39	88.6	11	25.0	33	75.0	5	11.4	39	88.6
	DT+dt	0	5	4.8	99	95.2	7	6.7	97	93.3	18	17.3	86	82.7	3	2.9	101	97.1
		1+	1	4.5	21	95.5	4	18.2	18	81.8	8	36.4	14	63.6	0	0.0	22	100.0
	FT + ft	0	4	8.9	41	91.1	2	4.4	43	95.6	6	13.3	39	86.7	2	44.4	43	95.6
		1+	2	2.5	79	97.5	9	11.1	72	88.9	20	24.7	61	75.3	1	1.2	80	98.8
4^th^-6^th^	Plaque	0	3	5.3	99	94.7	5	8.8	52	91.2	12	21.1	86	78.9	1	1.8	56	98.2
		1+	3	4.3	21	95.7	6	8.7	63	91.3	14	20.3	14	79.7	2	2.9	67	97.1
	Gingivitis	0	4	6.1	62	93.9	6	9.1	60	90.9	13	19.7	53	80.3	1	1.5	65	98.5
		1+	2	3.3	58	96.7	5	8.3	55	91.7	13	21.7	47	78.3	2	3.3	58	96.7
	Area of tongue coating	0,1	3	7.3	38	92.7	3	7.3	38	92.7	4	9.8	37	90.2	2	4.9	39	95.1
		2+	3	3.5	82	96.5	8	9.4	77	90.6	22	25.9	63	74.1	1	1.2	84	98.8
	Thickness of tongue coating	0,1	2	3.4	56	96.6	4	6.9	54	93.1	9	15.5	49	84.5	1	1.7	57	98.3
		2+	4	5.9	64	94.1	7	10.3	61	89.7	17	25.0	51	75.0	2	2.9	66	97.1
	DT+dt	0	3	3.7	78	96.3	16	19.8	65	80.2	10	12.3	71	87.7	0	0.0	81	100.0
		1+	1	3.2	30	96.8	5	16.1	26	83.9	5	16.1	26	83.9	0	0.0	31	100.0
	FT + ft	0	2	4.8	40	95.2	8	19.0	34	81.0	4	9.5	38	90.5	0	0.0	42	100.0
		1+	2	2.9	68	97.1	13	18.6	54	81.4	11	15.7	59	84.3	0	0.0	70	100.0
7^th^-9^th^	Plaque	0	0	0.0	23	100.0	5	21.7	18	78.3	6	26.1	17	73.9	0	0.0	23	100.0
		1+	4	4.5	85	95.5	16	18.0	73	82.0	9	10.1	80	89.9	0	0.0	89	100.0
	Gingivitis	0	0	0.0	43	100.0	6	14.0	37	86.0	8	18.6	35	81.4	0	0.0	43	100.0
		1+	4	5.8	65	95.5	15	21.7	54	78.3	7	10.1	62	89.9	0	0.0	69	100.0
	Area of tongue coating	0,1	2	2.9	66	97.1	12	82.4	56	82.4	10	14.7	58	85.3	0	0.0	68	100.0
		2+	2	4.5	42	95.5	9	79.5	35	79.5	5	11.7	39	88.6	0	0.0	44	100.0
	Thickness of tongue coating	0,1	2	2.4	82	97.6	14	16.7	70	83.3	12	14.3	72	85.7	0	0.0	84	100.0
		2+	2	7.1	26	92.9	7	25.0	21	75.0	3	10.7	25	89.3	0	0.0	28	100.0

## Discussion

Taste disorder was classified as the condition that expressed the impairment of taste sensation [3,9,10]. In this study, we defined taste hyposensitivity as the condition where the taste sensation was lower. This is the first study on taste hyposensitivity conducted for Japanese school students employing the four basic taste (sweet, salt, sour and bitter) tests. The whole-mouth taste test for 6–14 years old students revealed that 6.3% of them had sweet-taste, 14.3% had salt-taste, 20.9% had sour-taste and 6.0% had bitter-taste hyposensitivities. The prevalence of sweet- or salt-taste hyposensitivity was almost the same as that found in our former study targeted at high school students [11].

According to the another study, it was reported that the taste threshold declines in most of junior high school students [12]. Taste sensation is reported to become more sensitive with age in the study using an electrical taste test [13-15]. The current study presented similar results regarding sweet-, sour- and bitter-tastes, because the prevalence of taste hyposensitivity decreased with grade.

However, salt-taste showed a different tendency. The prevalence of salt-taste hyposensitivity was the lowest in 4^th^-6^th^ graders and the highest in 7^th^-9^th^ graders. The higher prevalence of salt-taste hyposensitivity found in junior high school students might be related to the diet, especially the amount of salt intake [15]. Our former study indicated that the frequent intake of isotonic drinks was associated with salt-taste hyposensitivity [11]. However, a nutrition survey was not carried out in this study, due to the time limitation of the school health education within the school curriculum. Further research including the nutritional investigation will be necessary in the future.

Many studies report that there is an association between taste sensitivity and zinc [3,16-19]. The actual amount of zinc in the body can be measured by a blood test. However, it is difficult to conduct a blood test in epidemiological studies, because the test is very invasive and the handling of large number of blood samples in schools is too complex to perform.

An analysis by gender found that the prevalence of bitter-taste hyposensitivity was higher in males than females. This result was similar to that in previous research, which reported a higher prevalence of women’s bitter-taste sensitivity than men [20].

Several studies reported that oral health status was associated with taste hyposensitivity in the elderly [21-24]. Satoh-Kuriwada *et al.* examined the taste of 18–31 years old university students by using the taste-disc method. The study revealed that 24.8% of subjects had a taste disorder and the alteration of taste sensitivity was related to dry mouth, tongue coating and damage or loss of taste buds [25].

However, a slight association was only observed between taste hyposensitivity and area of tongue coating in this study targeted school children. Further scrutiny of the relationship of taste disorder/hyposensitivity with oral health status will be necessary.

In this study, relatively large number of students in the 1^st^-3^rd^ grade presented sour or bitter-taste hyposensitivity. These generations of students might not recognize the sour- and bitter-taste correctly. One of the plausible reasons for this finding is that taste sensation does not have enough sensitivity, because the taste sensation may be not completely developed yet at this age. However this has not been demonstrated. It is necessary therefore to confirm the hypothesis biologically and continue research by performing sour- or bitter-taste test for younger grades of students.

There are various kinds of methods for taste tests, but which method is adequate for 1^st^-9^th^ grade students has not yet been established. The taste disc method is used to diagnose a taste disorder in individuals in a clinical environment, but it takes approximately 1 hour per person [7]. On the other hand, the whole-mouth method takes only 5 min per person, and it can be performed for many individuals at one time [7]. The whole-mouth method was performed in this study, because it is more efficient in screening taste hyposensitivity for a large group of people. In addition, it has the merit that students are able to recognize their own taste sensitivity. It is important to establish a standard method for the investigation of the taste hyposensitivity and to pursue the factors influencing taste hyposensitivity and its development. The whole-mouth method is considered the best candidate for epidemiological research on taste hyposensitivity.

Taste disorder/hyposensitivity is not the only dental problem. It also greatly influences dietary behavior, nutrition and systemic health [26,27]. As about 6.3-20.9% of students had hyposensitivity in this study, more detailed investigation of this problem would be necessary to improve their oral as well as general health.

## Conclusions

We conducted taste tests for four tastes using the whole mouth method, in schoolchildren aged 6–15 years. Taste hyposensitivity was observed in 6.0-20.9% of children. There was little association between taste hyposensitivity and oral health status. The current study implies that the factors affecting the taste hyposensitivity in children may different from those in the elderly. Therefore it is necessary to further investigate the causes of taste hyposensitivity among younger generation.

## Competing interests

The authors declare that they have no competing interests.

## Authors’ contributions

MO carried out the conception and design of the study, data acquisition, data analysis and draft the manuscript. MU participated in the conception and design of the study, data acquisition. TZ participated in the data acquisition. YK conceived of the study, and participated in its design and helped to draft the manuscript. All authors read and approved the final manuscript.

## Pre-publication history

The pre-publication history for this paper can be accessed here:

http://www.biomedcentral.com/1472-6831/14/36/prepub
